# Differential transcriptional response following glucocorticoid activation in cultured blood immune cells: a novel approach to PTSD biomarker development

**DOI:** 10.1038/s41398-019-0539-x

**Published:** 2019-08-21

**Authors:** Michael S. Breen, Linda M. Bierer, Nikolaos P. Daskalakis, Heather N. Bader, Iouri Makotkine, Mitali Chattopadhyay, Changxin Xu, Ariela Buxbaum Grice, Anna S. Tocheva, Janine D. Flory, Joseph D. Buxbaum, Michael J. Meaney, Kristen Brennand, Rachel Yehuda

**Affiliations:** 1 Department of Psychiatry, Icahn School of Medicine at Mount Sinai, New York, NY 10029 USA; 2 Department of Genetics and Genomic Sciences, Icahn School of Medicine at Mount Sinai, New York, NY 10029 USA; 3 Seaver Autism Center for Research and Treatment, Icahn School of Medicine at Mount Sinai, New York, NY 10029 USA; 4 Division of Traumatic Stress Studies, Icahn School of Medicine at Mount Sinai, New York, NY 10029 USA; 5 Mental Health Care Center, James J. Peters Veterans Affairs Medical Center, Bronx, NY 10468 USA; 6 Department of Psychiatry, McLean Hospital, Harvard Medical School, Belmont, MA 02478 USA; 7 Columbia Center for Translational Immunology, Columbia University Medical Center, New York, NY 10032 USA; 8 Friedman Brain Institute, Icahn School of Medicine at Mount Sinai, New York, NY 10029 USA; 9 Douglas Mental Health University Institute, McGill Group for Suicide Studies, McGill University, Montreal, Quebec H3A 0G4 Canada; 10 Ludmer Centre for Neuroinformatics and Mental Health, Department of Psychiatry, McGill University, Montreal, Quebec H3A 0G4 Canada; 11 Icahn Institute of Genomics and Multiscale Biology, Icahn School of Medicine at Mount Sinai, New York, NY 10029 USA; 12 Department of Neuroscience, Icahn School of Medicine at Mount Sinai, New York, NY 10029 USA

**Keywords:** Diagnostic markers, Psychiatric disorders, Comparative genomics, Biomarkers

## Abstract

Post-traumatic stress disorder (PTSD) is a condition of stress reactivity, whose clinical manifestations are evident when patients are triggered following exposure to a traumatic event. While baseline differences in gene expression of glucocorticoid signaling and inflammatory cytokines in peripheral blood mononuclear cells (PBMCs) have been associated with PTSD, these alterations do not fully recapitulate the molecular response to physiological triggers, such as stress hormones. Therefore, it is critical to develop new techniques that will capture the dynamic transcriptional response associated with stress-activated conditions relative to baseline conditions. To achieve this goal, cultured PBMCs from combat-exposed veterans with PTSD(+) (*n* = 10) and without PTSD(−) (*n* = 10) were incubated with increasing concentrations (vehicle, 2.5 nM, 5 nM, 50 nM) of dexamethasone (DEX). Across diagnosis and dosage, several genes and gene networks were reliable markers of glucocorticoid stimulation (FDR < 5%), including enhanced expression of *FKPB5*, *VIPR1*, *NR1I3*, and apoptosis-related pathways, and reduced expression of *NR3C1, STAT1*, *IRF1*, and related inflammatory and cellular stress-responsive pathways. Dose-dependent differential transcriptional changes in several genes were also identified between PTSD+ and PTSD−. Robust changes in expression were observed at 2.5 nM DEX in PTSD− but not PTSD+ participants; whereas, with increasing concentrations (5 nM and 50 nM), several genes were identified to be uniquely up-regulated in PTSD+ but not PTSD− participants. Collectively, these preliminary findings suggest that genome-wide gene expression profiling of DEX-stimulated PBMCs is a promising method for the exploration of the dynamic differential molecular responses to stress hormones in PTSD, and may identify novel markers of altered glucocorticoid signaling and responsivity in PTSD.

## Introduction

Biological studies of post-traumatic stress disorder (PTSD) have consistently pointed to hypothalamic-pituitary-adrenal (HPA) axis dysregulation and functional alterations of the glucocorticoid receptor (GR) as major contributors to the development and progression of the disorder^1–4^. Changes in GR sensitivity and alterations in peripheral blood gene expression profiles, including genes implicated in glucocorticoid signaling and inflammatory cytokine production – whether derived from a candidate^5^ or genome-wide exploratory approaches^6–8^—reflect some of the most robust laboratory findings for PTSD. However, it has been challenging to define the exact nature of these alterations and their functional implications because the HPA axis is a self-regulating system and glucocorticoid actions are binding site specific^9,10^. Moreover, since PTSD is a condition of stress reactivity, whose clinical effects are most evident when trauma survivors are triggered by the environment, there are likely distinct HPA axis alterations and gene expression responses associated with stress-activated conditions versus basal conditions. To date, knowledge of the molecular basis of PTSD has been limited by a lack of information regarding the dynamic response of gene expression to stress hormones in live, stimulated blood cells.

Early studies used dexamethasone (DEX) challenge tests in cultured peripheral blood mononuclear cells (PBMCs) from combat-exposed veterans with and without PTSD and observed that DEX-induced inhibition of lysozyme activity was greater in veterans with PTSD^11^. A significant correlation was observed between the lysozyme IC_50-DEX_ and the cortisol response to in vivo DEX administration in PTSD. Lipopolysaccharide-induced cytokine production from leukocytes, a measure of monocyte responsiveness, has also been reported to be more sensitive to DEX in combat veterans with PTSD compared to controls^4^. In studies of major depression, in vivo assessments of GR function have been conducted by analyzing gene expression profiles following DEX administration and report robust and reproducible changes in peripheral blood gene expression as well as decreased glucocorticoid sensitivity in depression^12–14^. Interestingly, DEX-induced gene expression revealed significantly increased *FKBP5* mRNA expression, which was dependent on *FKBP5* risk variants in depressed patients^15,16^.

There are, however, several intrinsic difficulties that accompany in vivo glucocorticoid challenges, specifically pharmacokinetic and individual variability in drug absorption, distribution, and metabolic profiles^17–19^. Moreover, glucocorticoid actions are highly dose-dependent^20–22^ and examination of genome-wide transcriptional responses across a broad range of concentrations of DEX would provide significantly greater information about the responsiveness of genes and gene networks to glucocorticoid stimulation in PTSD. It is for these reasons that in vitro GR challenge tests provide a complimentary and powerful framework to overcome complications of in vivo administrations. Nevertheless, there are no in vitro studies of glucocorticoid-induced gene expression in PBMCs, a critical gap that was recently highlighted in the literature^23^, and represents an important next step to measure HPA axis activity and GR function in PTSD patients.

Since glucocorticoid dysfunction is a well-replicated signature of combat-related PTSD, and glucocorticoids induce profound changes in gene expression, in vitro DEX-stimulation was expected to produce greater differences in gene expression patterns in PTSD than previously documented under basal conditions.

The primary goal of the current exploratory investigation was to compare DEX-stimulated changes in PBMC gene expression between combat-exposed veterans with and without PTSD. To this end, we developed a novel in vitro DEX dose-dependent challenge test and genome-wide RNA-sequencing data was generated from participant-derived PBMCs that were incubated with 0 nM (vehicle), 2.5 nM, 5 nM, and 50 nM of DEX. A multi-step analytic approach was used that specifically sought to identify: (1) DEX-stimulated genes and co-regulatory networks that change with increasing DEX concentrations; and (2) individual genes and networks that differ in response to DEX between PTSD cases and trauma-exposed controls, providing a basis for putative diagnostic biomarkers for the disorder.

## Materials and methods

### Participants and measures

Participants were combat-exposed veterans with and without PTSD *(n* = 10, respectively) who provided written, informed consent and for whom sufficient RNA for genome-wide expression analyses was extracted. Eligibility for participation was determined on the basis of a psychological evaluation using the Structured Clinical Interview for DSM-5 (SCID) and the Clinician Administered PTSD Scale (CAPS) for determination of PTSD diagnosis and severity^24,25^. Diagnostic and clinical exclusions included the presence of moderate or severe substance use disorder within the past 6 months, lifetime history of primary psychotic or Bipolar I disorders, neurological disorder or major systemic illness, and treatment with systemic steroids; for PTSD (−) only, current or recurrent major depressive disorder were exclusionary. Participants with self-reported history of moderate or severe traumatic brain injury (TBI) were excluded as this group is most likely to suffer long term cognitive problems related to the TBI. However, mild TBI (mTBI) is a very common historical exposure in veterans who served in Iraq and/or Afghanistan, referred to as the “signature injury” in this group of warfighters, and were included in the current study in order to lead to a study population that is well representative of OEF/OIF veterans. Notably, the majority of veterans exposed to a mTBI (loss of consciousness < 30 min) often do not report any observable cognitive problems beyond a few days/weeks following the injury. Participants stabilized on psychotropic medications were included. Participants also completed several self-report questionnaires including the Childhood Trauma Questionnaire (CTQ)^26^, which is comprised of 25 questions that ask individuals to record their impressions of childhood physical abuse, physical neglect, sexual abuse, emotional abuse and emotional neglect, using a 5-point Likert-type scale. Higher scores reflect higher levels of exposure to childhood adversity.

### Peripheral blood mononuclear cell isolation

Sixty ml of fasting blood was collected at 8 am by routine venipuncture in ethylenediaminetetraacetic acid (EDTA)-containing vacutainer tubes (VWR, West Chester, Pennsylvania). Platelet-rich plasma was separated by low-speed centrifugation at 120×*g* for 15 min at 22 °C. After collecting plasma, the remaining cells were diluted with an equal volume of Hank’s Balanced Salt Solution (HBSS; Gibco, Grand Island, New York) and PBMCs were isolated by density gradient centrifugation using Ficoll-Paque (GE Healthcare). The PBMC layer was collected, washed twice in HBSS and mononuclear cells were counted manually using a Cellometer Disposable Counting Chambers (Nexcelom Bioscience LLC. Lawrence, MA). The cells were re-suspended in complete RPMI, containing RPMI-1640 (Gibco), 10% fetal bovine serum, 50 U/ml penicillin–streptomycin mixture (Gibco) at a density of 1.75–2.00 × 10^6^ cells/ml of the medium.

### Dexamethasone treatment

Preliminary studies were conducted to identify optimal culture and glucocorticoid stimulation conditions for PBMCs. The current approach was based on our previously published functional assay, which measures the responsiveness to glucocorticoids^11^. In brief, the assay incubates PBMCs under 8 distinct doses (0.0, 0.5, 1.0, 2.5, 5, 10, 50, 100 nM) of DEX (Sigma-Aldrich) stock solution, which has high affinity to the glucocorticoid receptor. After a 3-day culture, lysozyme activity can be measured in the supernatant by turbidimetry using micrococcus lysodeikticus as a substrate. Since lysozyme synthesis is inhibited by DEX, reduction of lysozyme activity in our system reflects functional responsiveness to glucocorticoids (Fig. S1). Total RNA from the cell pellet of the culture from 14 subjects was used mRNA expression analysis using quantitative polymerase chain reaction (qPCR) experiments on several immune/glucocorticoid related genes (Fig. S1, B-J). Based on these results, doses 2.5 nM and 5 nM displayed the highest variance across genes and could be suitable as intermediate doses. For the selection of max dose, results indicated that 50 nM induces a max genomic response in all the samples.

Subsequently, 20 µl of DEX at concentration of 0, 27.5, 55, and 550 nM were pipetted in a flat bottom 96-well plate. To increase RNA yield, a total of 18 wells were prepared for each DEX concentration (~9.0 × 10^6^ cells per dose) in complete RPMI. PBMCs were prepared at 2.5 × 10^6^ cells/ml in complete RPMI and 200 µl were pipetted into each well, bringing the final concentration of DEX to 0, 2.5, 5, and 50 nM. Cultures were incubated at 37 °C, 5%_(vol/vol)_ CO_2_ for 72 hours. Following, the plates were centrifuged at 900×*g* for 15 min at 4 °C and 160 µl of the supernatant was collected and pooled from each DEX concentration well. For RNA isolation, the cell pellet on the bottom of each well was re-suspended in 100 µl of TRIzol reagent. Cell lysates for each DEX dose were pooled, aliquoted and stored at −80 °C until RNA isolation.

### RNA isolation, library preparation, and quantification of gene expression

RNA was extracted from TRIzol-lysed PBMCs using the miRNAeasy Mini Kit (Qiagen). RNA quantity was measured on the Nano Drop 2000 Spectrophotometer (Thermo Scientific) (56.6 ± 16.7 ng μl^−1^) and the quality and integrity measured with the Agilent 2100 Bioanalyzer (Agilent, Santa Clara, CA, USA). All RNA integrity numbers (RINs) were greater than 6 (RIN: 7.66 ± 0.79). The Illumina TruSeq Stranded Total RNA kit (Ilumina, San Diego, CA, USA) was used for library preparation accordingly to manufacturer instructions without any modifications. A total of 80 indexed RNA libraries were pooled and sequenced using long read paired-end chemistry (2 × 150 bp) at a read depth of 30 M reads per sample using the Illumina HiSeq2500. Adapter sequences were clipped and low quality reads were discarded using Trimmomatic^27^ using parameters: ILLUMINACLIP, MINLEN:140, CROP:140. All high-quality trimmed reads were then mapped to UCSC *Homo sapiens* reference genome (build hg37) using default STAR v2.4.0 parameters^28^ (percentage of mapped reads: 92.1% ± 1.6%). Samtools was used to convert bamfiles to samfiles and featureCounts^29^ was used to quantify gene expression levels for each individual sample using default paired-end parameters.

### Statistical analyses

**Fig. 1:**
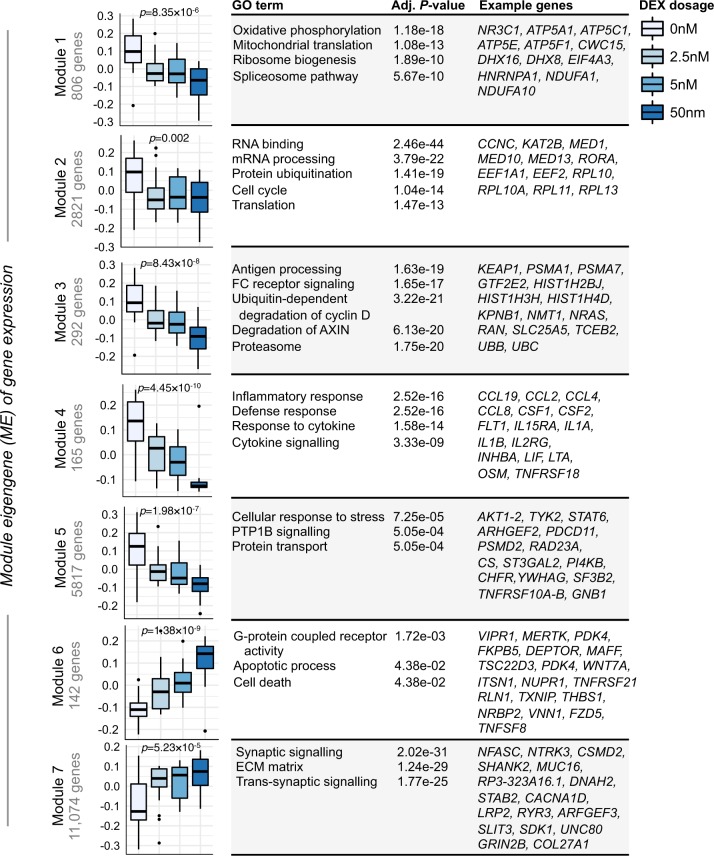
DEX-stimulated gene co-expression modules. Genes that were found to be significantly differentially expression between vehicle and 2.5 nM, 5 nM, and 50 nM of DEX (FDR < 5%), which were independent of PTSD status, were subjected to WGCNA analysis. A total of 21,117 genes were used as input. Seven modules were identified and an analysis of variance (ANOVA) was used to assess changes in module eigengene (ME) values with increasing concentration of DEX (*p*-values are labeled above each boxplot). Each module was subjected to gene ontology enrichment analysis and the top most significant enrichment terms and their associated Benjamini-Hochberg adjusted *P*-values are displayed. Further, we also display some of the top hub genes (kME > 0.6) within each module for quick interpretation of GR-stimulated gene co-expression modules and candidate individual genes.

Genome-wide RNA-sequencing data underwent extensive data pre-processing and quality-control, and normalized using the VOOM transformation method in the R package limma^30^. First, linear mixed effect models implemented through the R package varianceParition^31^ were applied to decompose the transcriptome into the percentage attributable to multiple biological and technical sources of variation. By properly attributing multiple sources of expression variation in this fashion, it is possible to identify and partially correct for some confounding variables. For each gene, the percentage of gene expression variation attributable to individual as a repeated measure (i.e., donor effects), RIN, BMI, DEX concentration, individual age, clinical diagnosis, childhood trauma exposure, and variation in basal immune cell type frequencies was computed. Second, differential gene expression analyses were conducted using a moderated *t*-test from the R package limma^30^. All analyses adjusted for the possible confounding influence of the following covariates: individual as a repeated measure, individual age, RIN, BMI, CTQ and basal cell type frequencies. In one instance (see Fig. 1), group status (PTSD+ versus PTSD−) was also included in the model to identify DEX-induced gene expression effects that were independent of PTSD status. Due to the repeated measures study design, where individuals are represented across distinct incubations of DEX (vehicle, 2.5 nM, 5 nM, 50 nM), the duplicateCorrelation function was applied in the limma analysis and gene level significance values were adjusted for multiple testing using the Benjamini and Hochberg method to control the false discovery rate. Third, unsupervised weighted gene co-expression network analysis^32^ was used to construct a signed co-expression network and identify DEX-stimulated changes in gene co-expression modules. Finally, all significantly differentially expressed genes and all network modules with genes passing intra-modular membership (kME) > 0.6 were subjected to functional annotation using the ToppFun module of ToppGene Suite Software^33^.

Full details on materials and methods are described in the SI Material and Methods.

## Results

### Clinical features and demographics: characterizing known sources of expression variation

Genome-wide RNA-sequencing profiles were generated from live cultured PBMCs incubated with increasing concentrations of DEX from a primary cohort of combat-exposed veterans with PTSD (PTSD+; *n* = 10) and without PTSD (PTSD−; *n* = 10) (Table S1). The two groups did not differ in age, gender, nicotine, race, prior-deployment and childhood trauma, but did differ in body mass index (BMI) and whether they screened positive for the Department of Veterans Affairs screening for possible traumatic brain injury (i.e., possible mTBI). To test the influence of various factors on gene expression profiles, for each gene, the percentage of gene expression variation attributable to each clinical and technical factor was computed (Fig. S2). Collectively, these variables explained ~40% of transcriptome variation, with donor as a repeated measure having the largest genome-wide effect that explained a median 8.7% of the observed variation, followed by RIN (3.9%), childhood trauma (3.7%) DEX concentration (3.7%) (Fig. S2, A–E). The remaining factors explained smaller fractions of overall transcriptome variation, including BMI (0.8%) and possible mTBI (<0.1%). Expression variation due to diagnosis (i.e., PTSD+ and PTSD−) had a detectable effect in a smaller number of genes. Genes with expression that varied most across different concentrations of DEX, included *STAT1-2*, *IRF1*, *IRF7*, *IFIT2-3* and transcription factor *ATF5*, as well as several other genes strongly implicated in cytokine and interferon signaling (Fig. S2, F–H). Notably, when samples were parsed by diagnosis, donor as a repeated measure explained a larger percentage of observed transcriptome variation within PTSD+ participants (12.2%) compared to PTSD- (5.5%; *p* = 2.2 × 10^−16^) (Figure S2,I), suggestive of a broader, more varied glucocorticoid response in PTSD+ PBMCs.

### Baseline differences in gene expression

Baseline gene expression profiles (vehicle; 0 nM DEX) were compared between PTSD+ and PTSD− groups while adjusting for the possible influence of donor as a repeated measure, age, prior-deployment, RIN, BMI, childhood trauma exposure, and basal cell type frequencies (see Supplemental Information). No significant differences in gene expression were observed (*q*-value < 0.05) and a distribution of PTSD-related *P*-values, which was no different from the expected uniform distribution, was identified (Fig. S3; λ mean = 0.69).

### DEX-stimulated genes and modules as markers of glucocorticoid activation

Dose-dependent transcriptional responses were examined following each increasing concentration of DEX relative to baseline and adjusted for the same variables as above. To focus on the dose-dependent effects of DEX and to identify reliable markers of glucocorticoid activation that are independent of PTSD status, an additional covariate of diagnosis (e.g., PTSD+ and PTSD−) was included into these analyses.

Relative to vehicle (0 nM DEX), a total of 1291, 15,197, and 19,439 genes were significantly differentially expressed (*q*-value < 0.05) following incubation with 2.5 nM, 5 nM, and 50 nM of DEX, respectively (Fig. S4); representing a total of 21,117 unique genes. To better understand the functional aspects of these DEX-stimulated changes in gene expression, the common unsupervised analytical tool weighted gene co-expression network analysis was applied to all 21,117 genes. Seven co-expression modules were identified and each displayed a unique module eigengene (*ME*) profile that significantly changed with increasing concentrations of DEX (Fig. 1, Table S2). ME values for modules 1–5 (M1–5) decreased in expression while ME values for modules 6-7 (M6-7) increased in expression. Modules M4 and M6 represent well-known reliable markers of glucocorticoid activation, and, compared to other modules, displayed clear dose-response effects. Module M4 decreased in expression and was found to be significantly enriched for processes related to inflammatory and cytokine signaling and harbored several relevant hub genes, including *CSF1*, *IL1B*, *IL1A*, *IL1RN*, *IL2RB, IL-2RG*, several chemokine ligands (e.g., *CCL2*, *CCL4*, *CCL8*) and transcription factors *ATF3*, *ATF5* and *MAFF*. Module M6 was increased in expression and implicated G-coupled protein receptor activity, apoptosis, and cell death related pathways. Module M6 also harbored several key hub genes, including *FKBP5*, *DEPTOR*, *PIK3IP1*, *NUPR1* and transcription factors *PER1 and RUNX2*. Interestingly, modules M4 and M6 also contain a significant fraction of genes with well validated glucocorticoid binding sites (*p* = 0.009, *p* = 0.001, respectively), which are known to have significant glucocorticoid-inducible gene regulatory activity^10^ (Fig. S5).

Module M1 decreased in expression and was implicated in oxidative phosphorylation and mitochondrial translation and included the glucocorticoid receptor gene, *NR3C1*. Modules M2, M3, and M5 were also downregulated were respectively enriched for translation, ubiquitin-proteasomal pathway, and protein transport, representing pathways, which have been previously reported to be downregulated with low doses of glucocorticoids across various experimental contexts.

Finally, no module showed distinct differences in co-regulatory patterns between PTSD+ and PTSD− groups (Figure S6), indicating that lack of a clear dose-response is not confounded by PTSD status.

### Differential transcriptional responses to DEX in PTSD

**Fig. 2:**
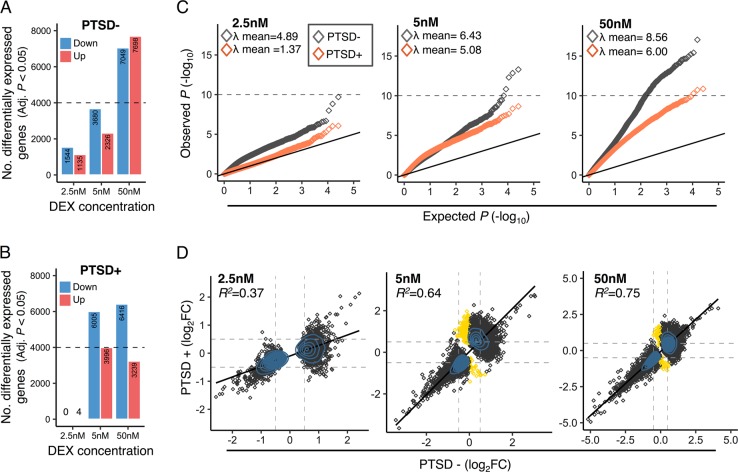
Differential transcriptional response to DEX in PTSD. DEX-stimulated changes in gene expression were evaluated separately for PTSD− and PTSD+ participants. A clear distinction for gene expression changes that were either suppressed or enhanced for DEX were identified for **a** PTSD− and **b** PTSD+ participants. **c** Quantile-quantile (QQ)-plots demonstrate an observed distribution of *p*-values, which greatly deviates from the expected uniform distribution across all three concentrations of DEX. The genomic inflation factor (λ, also defined as median *X*^*2*^) was computed to measure deviations of the observed genome-wide distribution of the test statistic from with the expected null distribution. A mean λ of 1 indicates no difference from the expected null distribution, while λ > 1 indicates marked shifts from the expected null distribution. **d** Concordance of genome-wide log2 fold-changes for all differentially expressed genes (Adj. *P* < 0.05) were computed in a dose-dependent manner and a linear regression model assessed the overall correspondence between PTSD− and PTSD+ participants. Gold points indicate gene expression changes that are unique to PTSD+ participants. Blue shading indicates a density distribution, whereby an excess of data points are depicted by a denser shading.

Next, the dose-dependent transcriptional response to DEX was assessed separately in PTSD+ and PTSD- participants (Table S3). At 2.5 nM of DEX, PTSD− participants displayed 1544 downregulated genes and 1135 upregulated genes (*q*-value < 0.05), while PTSD + participants displayed four up-regulated genes and no single gene was downregulated (Fig. 2a, b). With increasing concentrations of DEX, 2326 genes were up-regulated and 3680 genes were downregulated in PTSD− participants following stimulation with 5 nM of DEX, whereas 7698 genes were up-regulated and 7049 genes down-regulation following stimulation with 50 nM of DEX (Fig. 2a). A significant proportion of DEX-stimulated changes in gene expression was found in common between 5 nM and 50 nM DEX in the PTSD− group ( ∩ = 5484, OR = 21.3 *p* < 2.0 × 10^−16^) (Fig. S7). In parallel, PTSD+ group displayed upregulation of 3996 genes and down-regulation of 6516 genes with 5 nM of DEX, and with 50 nM of DEX, 3239 genes were up-regulated and 6516 genes were down-regulation. Similarly, a substantial fraction of these DEX-stimulated changes in gene expression overlapped between 5 nM and 50 nM DEX for PTSD+ participants ( ∩ = 6818, OR = 15.9 *p* < 2.0 × 10^−16^) (Fig. S7). Importantly, compared to the *p*-value distributions derived from baseline gene expression analysis, quantile-quantile (QQ)-plots demonstrate an observed distribution of *p*-values, which greatly deviates from the expected uniform distribution across all three concentrations of DEX, whereby PTSD- participants displayed a larger fraction of genes with an abundance of low *p*-values (Fig. 2c). To further quantify these results, the concordance between directionality of change statistics (log fold-change) was measured for the DEX-stimulated genes in PTSD- and PTSD+ groups in a dose-dependent manner (Fig. 2d). Low-to-moderate levels of concordance were observed for DEX-stimulated gene expression profiles at 2.5 nM (*R*^*2*^ = 0.37), which strengthened with increasing concentrations to 5 nM and 50 nM DEX (*R*^*2*^ = 0.64, *R*^*2*^ = 0.75, respectively).

**Fig. 3:**
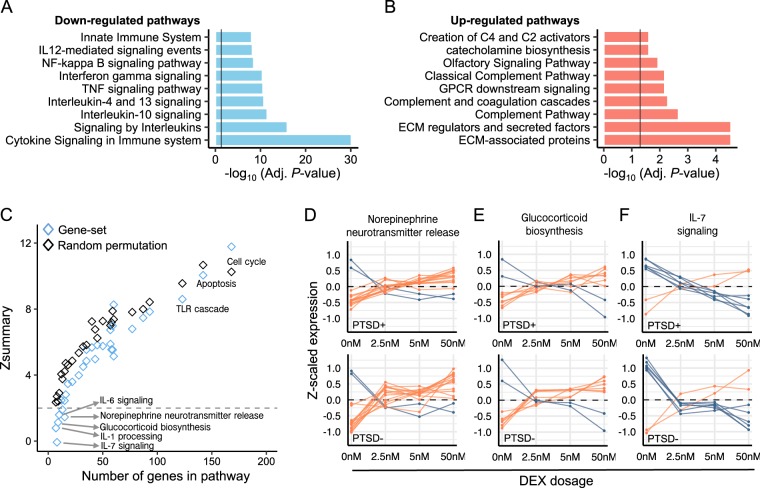
Differential response to DEX within functional gene sets. Gene ontology enrichment was performed on the significantly differentially expressed genes (Adj. *p* < 0.05) from vehicle to 2.5 nM DEX in PTSD- participants and were parsed by (**a**) downregulated genes and (**b**) up-regulated genes. (**c**) Gene set preservation analysis was performed on all gene sets with significant enrichment results (Table S4) to identify gene sets with the most differential response to DEX between PTSD− and PTSD+ participants. Randomly selected groupings of genes matching the same number of genes within each gene set were also permuted to provide n preservation-based estimate of what is expect by chance. Six gene sets displayed no preservation (Z_summary_ < 2) between PTSD+ compared to PTSD- participants. **d**–**f** Z-scaled expression data examines the average expression profiles across vehicle, 2.5 nM, 5 nM, and 50 nM concentrations of DEX for three gene sets with differential responses to DEX, including **d** norepinephrine neurotransmitter release, **e** glucocorticoid biosynthesis and **f** IL-7 signaling. Increased response at 2.5 nM of DEX was observed for norepinephrine neurotransmitter release and glucocorticoid biosynthesis while decreased response at 2.5 nM DEX was observed for IL-7 signaling. Red lines indicate genes that increase with expression and blue lines indicate genes that decrease in expression. Dots represent averages across all samples.

Genes that were uniquely and significantly (*q-*value < 0.05) downregulated in the PTSD- group following 2.5 nM of DEX were strongly enriched for several molecular pathways, including cytokine signaling, signaling by interleukins and TNF signaling pathways (Fig. 3a), while genes that were uniquely and significantly upregulated were implicated in the complement pathway, GPCR signaling and ECM-associated genes/proteins (Fig. 3b, Table S4). As these biological processes demonstrate significant differential response in PTSD following low doses of DEX, these gene sets were further interrogated for differential responses following higher doses of DEX using gene set preservation analysis (see Supplemental Information). While most gene sets did not show significant differential responses to increasing concentrations DEX and were highly preserved between PTSD- and PTSD+ participants (e.g., cell cycle, apoptosis, TLR cascades), several other gene sets showed differential transcriptional responses to DEX between PTSD+ and PTSD- participants, including norepinephrine neurotransmitter release, glucocorticoid biosynthesis and IL-1, IL-6 and IL-7 signaling pathways (Z_summary_ < 2) (Fig. 3c, Table S5). Genes mapping to these gene sets revealed marked differences in expression profiles following 2.5 nM, 5 nM and 50 nM of DEX between PTSD- and PTSD+ participants (Fig. 3d–f).

### DEX-induced changes in glucocorticoid regulatory genes

**Fig. 4:**
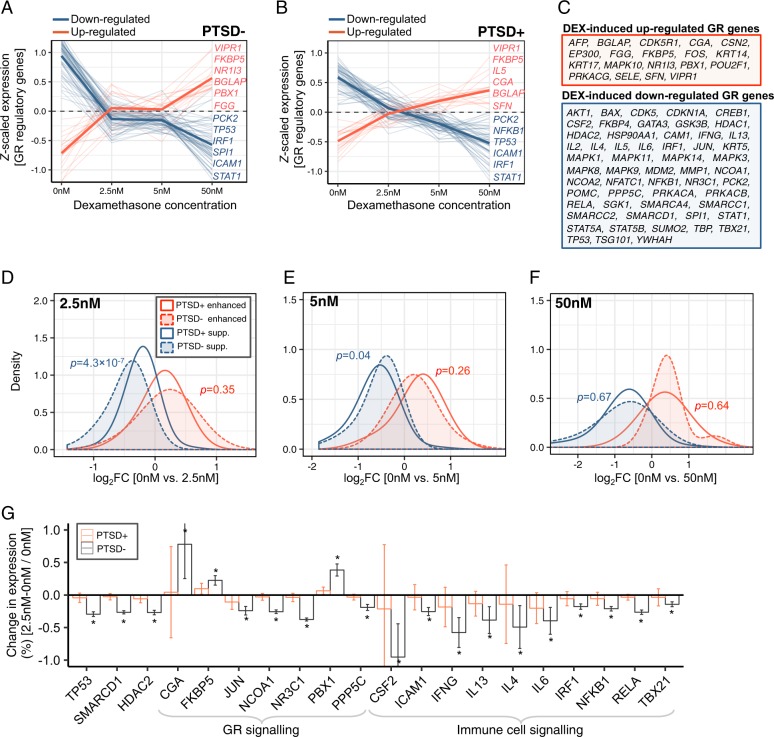
DEX-stimulated effects on glucocorticoid regulatory genes. We examined the effects of DEX on a curated list of 75 well-known glucocorticoid regulatory genes. A clear distinction for gene expression changes that were either suppressed or enhanced for DEX were identified for **a** PTSD− and **b** PTSD + participants. Dark solid lines indicate average splines across all DEX-induced increased (red) and decreased (blue) genes. **c** A total of 19 genes were consistently enhanced by DEX while 55 genes were consistently suppressed by DEX. Difference in the magnitude of gene for DEX-suppressed and –enhanced genes were evaluated for PTSD− and PTSD+ participants **d** at 2.5 nM, **e** 5 nM, and **f** 50 nM of DEX. A Wilcox-rank sum test was used to compare differences in distribution of log_2_ fold-changes (FC) between groups. **g** GR-related genes that were significantly (Adj. *p* < 0.05) and uniquely differentially expressed in PTSD- participants following 2.5 nM of DEX. A full list across all dosages can be found in Table S6.

Subsequently, our exploratory investigation focused on a well-curated list of 75 GR regulatory network genes, which have been the intense focus of GR activation and PTSD biomarker research. These genes were collected through the Pathway Interaction Database (set no. M115) and then were manually examined to ensure that only GR-related genes were included within the final list. Gene expression levels for these GR-related genes were examined for PTSD-related differential response to DEX, and revealed that GR-related genes were either strongly up-regulated or downregulated by DEX (Fig. 4a, b, Table S6). A total of 19 GR-related genes were found to be significantly increased in response to DEX in both PTSD− and PTSD+ participants, including genes *FKPB5*, *NR1I3*, *VIPR1*, *PBX1*, and *FGG* (Fig. 4c). The remaining 55 genes were found to be downregulated by DEX in both PTSD- and PTSD+ participants, including genes *STAT1*, *ICAM1*, *IRF1*, and *TP53* (Fig. 4c). Notably, *IL-5* gene expression displayed a difference in response to DEX whereby expression increased for PTSD+ participants and decreased for PTSD− participants.

Subsequently, we examined these genes, which were increasing and decreasing in expression following DEX treatment, for significant differences in their magnitude of change between diagnostic groups. Of the 55 DEX induced downregulated GR-related genes, a greater decrease was observed for PTSD- compared to PTSD+ participants at the lowest treatment dose (2.5 nM of DEX) (*p* = 4.3 × 10^−7^) (Fig. 4d), particularly for glucocorticoid receptor *NR3C1*, *IFN-γ*, *IL4*, *IRF1*, transcription factor *JUN* and others (Fig. 4g). No significant differences in overall levels of DEX-induced change in gene expression were observed in GR-related between PTSD+ and PTSD− participants following treatment with higher treatment doses (5 nM and 50 nM of DEX) (Fig. 4e, f).

### RT-qPCR validation of differential responses to DEX

**Fig. 5:**
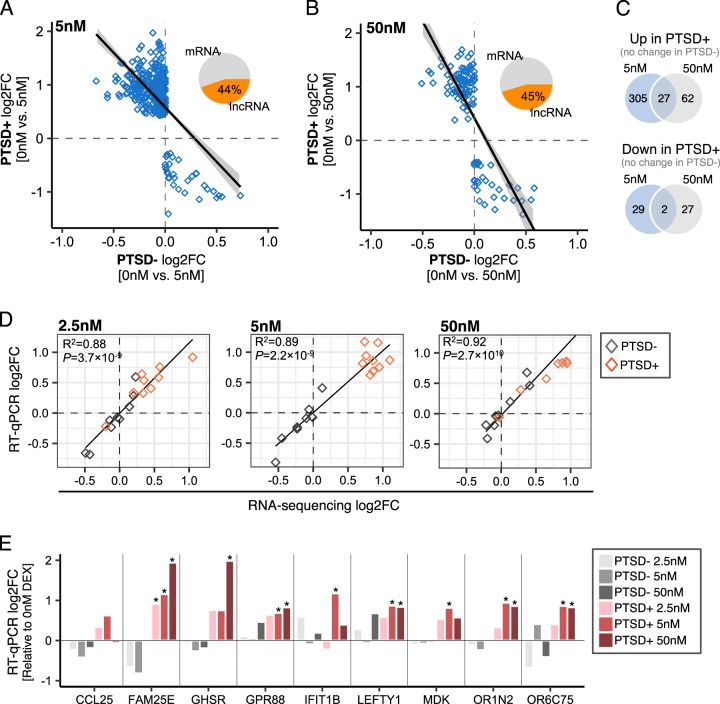
Validating differential responses to DEX in PTSD+ participants. **a** A total of 363 genes were significantly and uniquely responsive following 5 nM of DEX and **b** a total of 118 genes were uniquely responsive following 50 nM of DEX in PTSD+ participants. **c** Overlap analysis of DEX stimulated genes following 5 nM and 50 nM. **d** Real-Time quantitative PCR (RT-qPCR) was used to validate top performing mRNA targets that were uniquely upregulated in PTSD+. For these genes, the concordance between log2 fold-change statistics was assessed using a linear regression model between RT-qPCR and RNA-sequencing results for PTSD+ (red) and PTSD- (gray) participants. **e** Log2 fold-changes for RT-qPCR results validate a unique transcriptional response to PTSD+ participants. Asterisks (*) indicate changes that are significantly different from vehicle and were computed using a moderated *t*-test.

Under close inspection, 332 genes were uniquely upregulated and 31 genes were uniquely downregulated in PTSD+ participants following 5 nM of DEX (*q*-value < 0.05) (Fig. 5a, shaded in gold in Fig. 2d). In response to 50 nM of DEX, 89 genes were uniquely upregulated and 30 genes were uniquely downregulated in PTSD+ participants (Fig. 5b). A significant fraction of genes that were uniquely upregulated following 5 nM of DEX were also upregulated following 50 nM exposures of DEX ( ∩ = 27, OR = 17.8 *p* = 5.0 × 10^–33^) (Fig. 5c). These genes included *CCL25*, *FAM25E*, *GHSR*, *GRP88*, *IFIT1B*, *MDK*, O*RIN2*, *O6C75*, and *LEFTY* and several lncRNAs (~45%) (Table S7). We validated these nine genes displaying differential response to DEX in a subset of samples by RT-qPCR (see Supplemental Information). Strong concordance was observed for directionality of change statistics (log fold-change) between RT-qPCR results and RNA-sequencing results for these eight mRNAs at 2.5 nM (*R*^*2*^ = 0.88), 5 nM (*R*^*2*^ = 0.89) and 50 nM (*R*^*2*^ = 0.92), indicating strong independent technical validation (Fig. 5d). Furthermore, these RT-qPCR targets also validate the significant differences identified using RNA-seq between PTSD+ and PTSD− participants at 2.5 nM and 50 nM of DEX (Fig. 5e).

## Discussion

Here, we report the first demonstration of genome-wide gene expression effects in response to in vitro glucocorticoid treatment in PTSD. By exposing cultured PBMCs to increasing concentrations of the synthetic glucocorticoid DEX and examining genome-wide transcriptional responses, our findings capture the dynamic response of gene expression to glucocorticoid stimulation and reveal several novel findings of glucocorticoid function and responsivity in PTSD. First, these results illustrate that different concentrations of DEX activate very different genes and gene networks, illuminating a well-known, but not often acknowledged the complexity of dose-dependent glucocorticoid effects. Second, DEX-stimulated changes in gene expression achieve enhanced effect sizes, which are required to leverage gene expression profiles as actionable, clinical biomarkers for PTSD, even with limited sized samples. Finally, these findings illustrate that in vitro stimulation of blood immune cells with DEX permits for a more effective discrimination between PTSD+ and PTSD− participants than baseline gene expression with the potential for discovery of PTSD biomarkers. All of these points are discussed in turn below.

DEX-responsive gene co-expression modules were identified indicating widespread effects of DEX on diverse cellular signaling systems, many of which have been linked to glucocorticoid activity and/or PTSD. The gene modules with the most distinct dose-response to DEX were modules M4 and M6. Module M4 decreased in expression and was enriched for processes related to the inflammatory response and cytokine signaling while M6 increased in expression and was implicated in apoptosis-related processes. Both modules contained genes harboring a significant fraction of glucocorticoid binding sites with glucocorticoid-inducible gene regulatory activity. After ligand binding, the GR trans-locates to the nucleus where it binds to glucocorticoid binding sites and glucocorticoid response element sequences in genes resulting in transactivation^10^. Indeed, such glucocorticoid-induced transactivation has been mechanistically linked to the induction of pro-apoptotic genes^34,35^, however, ligand binding to the GR also results in the down-regulation of inflammatory and cytokine signaling genes^36^, which likely explains the clear DEX-induced dose-responses in modules M4 and M6. Decreases in PTP1B signaling and protein transport (M5) were also observed, and previous research has identified pro-inflammatory cytokines, such as TNF-α, as positive regulators of *PTP1B* expression in diverse cell lines and tissues^37^. Likewise, reduced expression of oxidative phosphorylation and mitochondrial translation (M1) was also observed whereby similar investigations have reported reduced efficiency of mitochondrial oxidative phosphorylation in response to low doses of DEX across various experimental conditions and tissues^38^. Similar reports have described the inhibitory effects of DEX on protein-ubiquination (M2) and cyclin D and axin expression (M3), that occur on a component of the mitogen signaling cascade parallel to the ERK pathway^39^. Together, these results highlight both the immuno-suppressive and -enhancing effects of glucocorticoids on a number of cellular signaling cascades.

Moreover, while several molecular pathways and gene sets display strong responses to DEX, there are several genes within these gene sets with either minimal or no change in expression. For example, while a significant fraction of inflammatory and cytokine signaling genes were robustly downregulated with increasing concentrations of DEX, a substantial fraction of these was genes also unchanged in expression, including C-reactive protein (*CRP*), *IFNA2*, *IL5*, *IL9*, *IL20*, several chemokine ligands (e.g*.*, CCL11, *CCL21*) and chemokine receptors (e.g*., CCR3*, *CCR7*). Similarly, while several apoptosis-related genes were up-regulated, others also were unchanged in gene expression, including *AIFM3*, *BCL2L10*, *CREBBP*, *ERBB2*, several caspases (e.g*.*, CASP2, *CASP6*). Follow-up functional studies integrating additional unbiased genome-scale data, including DNA methylation and ChIP-Seq are likely required to fully clarify these differences.

The current study further demonstrates that in vitro stimulation of blood immune cells via DEX significantly amplifies gene expression effect sizes into a range which may facilitate actionable, clinical biomarkers for PTSD. Small sample sizes have almost universally been inadequate for biomarker discovery, especially in the context of cross-sectional post-trauma baseline gene expression studies of PTSD, which have consistently produced small effect sizes^6–8^. Further, similar results were also derived from the largest PTSD peripheral blood transcriptomic study conducted to date (Fig. S4, A-B)^7^, and provide additional support for the weak effects observed at baseline (vehicle; 0 nM) between PTSD− and PTSD+ participants in the current study. In fact, current sample size estimates indicate that >5000 samples are required to reach clinically meaningful effect sizes (power = 0.8) for blood-based gene expression biomarker discovery in PTSD^7^. The lack of robust changes in gene expression and the equivocal results produced from baseline peripheral blood gene expression profiles in PTSD are not surprising, given that baseline measurements of neuroendocrine markers have also not been as well replicated as those that have involved the response to glucocorticoid challenge, such as the low dose dexamethasone suppression test^40–42^. The ability to examine the dynamic gene expression response to provocation not only provides more textured and relevant information about PTSD, it may increase reproducibility across studies. Indeed, PTSD is a disorder of stress reactivity, most often observed clinically when patients are triggered. The results underscore the need to develop new measures for examining transcriptional responses immediately following glucocorticoid activation. Moreover, the data presented here provide an important proof-of-principle approach and show that DEX-stimulated gene expression changes in an in vitro preparation of blood immune cells permits for a more powerful discrimination between PTSD+ and PTSD− participants than does examination of gene expression levels of quiescent ex vivo PBMCs.

Our genome-wide approach has also highlighted several dose-dependent differences in the transcriptional response to DEX in PTSD. The dose-response relation between DEX concentration and transcriptional activity is consistent with previous pharmacological studies as well as with the known affinity (~5 nM) of DEX for the GR receptor^43^. Moreover, these data indicate a weaker glucocorticoid-induced transcriptional response in PTSD+ relative to PTSD− following a low dose of DEX (2.5 nM) suggesting enhanced functional ‘sensitivity’ to DEX. This striking group difference was absent at higher doses. It is important to note that the term ‘sensitivity’ is operationally defined. Glucocorticoids can affect transcription through multiple mechanisms not all of which involved direct GR binding to DNA (e.g*.*, protein-protein interactions). However, the emergence of the difference in DEX-induced transcription at a concentration well within the range of the Kd of DEX for the GR is consistent with a GR-mediated effect. Likewise, both co-expression modules (Fig. 1) and multiple gene sets (Fig. 3d) reveal clear dose (or concentration) – response functions reflecting a primary effect at 2.5 nM DEX.

The findings of increased DEX-related GR ‘sensitivity’ of PMBCs from the PTSD- contrasts with previous research from our group. Our previous research revealed that trauma-exposed combat veterans with and without PTSD displayed a similar density of GR in leukocytes, but only combat veterans with PTSD showed a decrease in leukocyte GR number following low dose DEX challenge^1–3,44^. A higher GR-‘sensitivity’ in PTSD was also observed following DEX-induced inhibition of lysozyme activity in monocytes^4^. This discrepancy should not be surprising. Glucocorticoid effects on both DNA binding, as revealed in ChIP-seq analyses, and transcriptional regulation are highly tissue specific^45^. DNase hypersensitivity assays, which reflect chromatin accessibility, together with GR ChIP sequencing reveal striking differences in both targets and transcriptional effects across cell types^46^. Tissue-specificity may thus be conferred by diversity in chromatin accessibility as well as by multiple tissue-specific modulators of glucocorticoid action (e.g*.*, FKBP5, 11ßHSD 1 or 2, etc.) or tissue-specific expression of GR variants (i.e., GRa and GRß) that can have opposing effects^47^. Differences in tissue responses to glucocorticoids may be mediated by tissue-specific group differences at multiple levels. Likewise, environmental regulation of the methylation status of the GR gene is similarly tissue-specific^48^. Additionally, GR regulation of transcription is also determined by the form of GR binding to DNA. GR’s bind DNA as monomers or homodimers, with resulting differences in transcriptional effects. Finally, the GR effects on transcription are also regulated by cellular context: GR’s interact with the binding of other transcription factors (e.g*.*, AP-1, *CREB*, etc.) with DNA, which can alter transcriptional effects^45^. In sum, tissue-specific differences in GR ‘sensitivity’ between PTSD + and PTSD- subjects are both unsurprising and a highly attractive target for molecular analysis.

The transcriptional responses captured by stimulating cultured PBMCs with DEX, and discussed here, provide interesting candidates for further glucocorticoid and/or PTSD studies. For example, the identification of several lncRNAs with differential transcriptional responses to DEX in PTSD+ participants align with a growing body of literature linking ncRNAs with key aspects of PTSD pathophysiology^49^. Therefore, a potential application for this type of work is the identification of genes and molecular processes that are highly and differentially influenced by glucocorticoids. Towards this end, and to promote the exchange of this information, we developed a web application with an easily searchable interface to act as a companion to this paper and is available from the following URL, https://breenms.shinyapps.io/DEXPTSD/. Using the application, researchers can quickly query any gene of interest to evaluate the dose-dependent effect of DEX on gene expression levels.

This study also presents some limitations. First, these results require replication and further follow-up in larger independent cohorts composed of mixed biological sexes that have been well-characterized clinically. However, it is worth noting that while the majority of baseline PBMC transcriptome investigations in PTSD, including our own large-scale transcriptome work^7^, produce gene level results with weak effect sizes (Fig. S4), these preliminary results are novel in demonstrating robust group differences in response to DEX even among a small sample (i.e*.*, some aspects of a stress-related disorder may only be observable under conditions of elevated arousal/stress). Second, it is likely that future work examining such transcriptional responses at an individual single cell level in a well-defined, genotyped population will further establish these methods while uncovering a landscape of PTSD biomarkers that will serve as definitive guides for the study of PTSD stress responsivity, resiliency and vulnerability. Third, it will be important to relate the panel of transcriptional findings to neuroendocrine and in vitro measures of GR sensitivity that have been well studied in trauma-exposed samples. Finally, clarifying and testing the relationships between DEX-responsive genes in cultured peripheral blood samples versus patient-derived neuronal samples, will help to inform new treatment targets and the development of next-generation mechanism-based therapeutics.

In sum, these results provide support for the utility of glucocorticoid-stimulated gene expression profiles as potent tools for the study of PTSD pathophysiology, for the detection of target treatments, and for the development of blood biomarkers for PTSD-related GR sensitivity. With replication and validation, an ultimate application of this preliminary work is to provide tools that can serve as a resource to stimulate and enable additional studies to further elucidate the complex transcriptional response to stress hormones and, in turn, facilitate the development of PTSD biomarkers.

## Supplementary information

Supplemental Information

Supplemental Figures 1-7

Supplemental Tables 1-7
