# Correction: Differential transcriptional response following glucocorticoid activation in cultured blood immune cells: a novel approach to PTSD biomarker development

**DOI:** 10.1038/s41398-019-0665-5

**Published:** 2020-01-06

**Authors:** Michael S. Breen, Linda M. Bierer, Nikolaos P. Daskalakis, Heather N. Bader, Iouri Makotkine, Mitali Chattopadhyay, Changxin Xu, Ariela Buxbaum Grice, Anna S. Tocheva, Janine D. Flory, Joseph D. Buxbaum, Michael J. Meaney, Kristen Brennand, Rachel Yehuda

**Affiliations:** 1 Department of Psychiatry, Icahn School of Medicine at Mount Sinai, New York, NY 10029 USA; 2 Department of Genetics and Genomic Sciences, Icahn School of Medicine at Mount Sinai, New York, NY 10029 USA; 3 Seaver Autism Center for Research and Treatment, Icahn School of Medicine at Mount Sinai, New York, NY 10029 USA; 4 Division of Traumatic Stress Studies, Icahn School of Medicine at Mount Sinai, New York, NY 10029 USA; 5 Mental Health Care Center, James J. Peters Veterans Affairs Medical Center, Bronx, NY 10468 USA; 6 Department of Psychiatry, McLean Hospital, Harvard Medical School, Belmont, MA 02478 USA; 7 Columbia Center for Translational Immunology, Columbia University Medical Center, New York, NY 10032 USA; 8 Friedman Brain Institute, Icahn School of Medicine at Mount Sinai, New York, NY 10029 USA; 9 Douglas Mental Health University Institute, McGill Group for Suicide Studies, McGill University, Montreal, Quebec H3A 0G4 Canada; 10 Ludmer Centre for Neuroinformatics and Mental Health, Department of Psychiatry, McGill University, Montreal, Quebec H3A 0G4 Canada; 11 Icahn Institute of Genomics and Multiscale Biology, Icahn School of Medicine at Mount Sinai, New York, NY 10029 USA; 12 Department of Neuroscience, Icahn School of Medicine at Mount Sinai, New York, NY 10029 USA

**Keywords:** Diagnostic markers, Psychiatric disorders, Comparative genomics, Biomarkers

**Correction to:Translational Psychiatry**

10.1038/s41398-019-0539-x published online 21 August 2019

This Article was originally published without the correct Supplemental Table file (Table S1 was missing). In total, there are seven Supplemental Tables, and six were in the original submission. Furthermore, Fig. 1 was misplaced in the main text; it was embedded in the manuscript file even before the results section. Both issues have now been fixed in the HTML and PDF versions of this Article.

