# Supplementary material for: Differential transcriptional response following glucocorticoid activation in cultured blood immune cells: a novel approach to PTSD biomarker development
Source: Transl Psychiatry. 2019 Aug 21;9:201. doi: 10.1038/s41398-019-0539-x (PMC6704073; doi:10.1038/s41398-019-0539-x)
Supplement: Supplementary file 1 — Supplemental Information [file 41398_2019_539_MOESM1_ESM.docx]

### Data pre-processing

### Raw count data measured 56,277 transcripts across 80 samples. Non-specific filtering removed genes with zero counts and lowly expressed genes that did not meet the requirement of a minimum of one count per million (cpm) in at least five samples. A total of 38,702 transcripts were retained and then subjected to limma VOOM normalization (1), a variance-stabilization transformation method. Normalized data were inspected for outlying samples using unsupervised hierarchical clustering of subjects (based on Pearson coefficient and average distance metric) and principal component analysis (PCA) to identify potential outliers greater than two standard deviations from these averages. A total of five outliers were present in these data and resulting normalized values were used as input for downstream analyses.

### Immune cell type frequencies at baseline

### Complete blood counts were obtained at the time of blood collection for each participant that allowed for testing of associations with estimated circulating immune cell type frequencies based on gene expression profiles at baseline (0nM DEX). In order to predict the proportions of immune cell types based on baseline (0nM) gene expression profiles, the Cibersort cell type de-convolution tool was used (https://cibersort.stanford.edu/) (2). Cibersort relies on known cell subset specific marker genes and applies linear support vector regression, a machine learning approach that is highly robust compared to other methods with respect to noise, unknown mixture content, and closely related cell types. As input, we used the LM22 signature matrix to distinguish four main leukocytes subtypes: B cells, T cells (CD3+), natural killer (NK) cells (CD56+), monocytes (CD14+). The LM22 matrix can be further divided into 18 less frequent immune cell subsets, which we pooled and defined as ‘other’. Pearson correlation coefficients were computed between the percentage of differential blood counts and predicted immune cell type frequencies at baseline (0nM DEX) and overall high levels of concordance were observed for each major immune cell type (*R*=0.81, B cells; *R*=0.83, CD4+; *R*=0.79, CD14+; *R*=0.89, CD56+ cells). These high levels of concordance supported the inclusion of observed immune cell type frequencies at baseline as a covariate in differential gene expression analyses in order to adjust for potential expression differences due to basal cell type frequencies.

### Differential gene expression analysis

### Since genome-wide gene expression profiles may be influenced by differences in technical, biological and clinical factors, a linear mixed effect model was applied to decompose the computed transcriptome into the percentage attributable to such sources of variation using the R package varianceParition (3). This approach quantified the main sources of variation in the expression dataset attributable to differences in RIN, BMI, DEX concentration, individual age, individual as a repeated measure, clinical diagnosis, childhood trauma (as estimated by the Childhood Trauma Questionnaire (CTQ)) and basal immune cell type proportions. Sustained childhood trauma, rather than the presence or absence of a PTSD diagnosis, has previously been linked with hyper-responsiveness of the HPA axis and warrants its inclusion as a covarate (4). These variables explained ~40% of transcriptome variability, with individual having the largest genome-wide effect, explaining a median 8.7% of the observed variability (Figure S1). By attributing gene expression patterns to multiple sources of variation in this fashion, it is possible to identify and partially correct for some variables. Subsequently, all differential gene expression analysis were conducted using a moderated *t*-test from the R package limma (1) covarying for the following variables: individual as a repeated measure, RIN, BMI, age, CTQ total score, and baseline estimated cell type proportions. For one analysis (*i.e.*, Figure 1), diagnostic group was also entered as a covariate in order to identify genes and networks that are dynamically regulated by DEX, independent of PTSD diagnosis. The duplicateCorrelation in limma was used to account for measuring multiple samples per donor and gene level significance values were adjusted for multiple testing using the Benjamini and Hochberg method to control the false discovery rate (FDR). Genes passing a multiple test corrected *P*-value < 0.05 were labeled significant.

### To systematically evaluate the influence of each covariate on our linear model, we also examined the genome-wide concordance of β-statistics derived from each model as we consecutively added the next leading covariate to the model (followed by next leading covariate, and so on) until the full model was achieved. We observed that that the influence of individual as a repeated measure and RIN had the largest effect on the model (as predicted from our variancePartition analysis), whereas the inclusion of the all subsequent covariates had very little overall influence and do not greatly effect gene-level results. It is also worth mentioning that we use the limma software to fit a linear model to the gene expression data and as long as one has enough residual degrees of freedom for variance estimation, this method will still work in terms of controlling type I error. Of course, unnecessary increases to the complexity of the model could also reduce power as the estimates of the model parameters may become more uncertain if the additive factors are overwhelmingly complex. Nevertheless, based on our results, we did not find strong evidence for the additional factors in the model to influence our final results.

### Genomic inflation factor, lambda

### Computing the genomic inflation factor (λ) with quantile-quantile (QQ)-plots offers a statistical approach to quantify and visualize marked deviations of the observed genome-wide distribution of the test statistic from the expected null distribution. This statistic is conventionally used in genome-wide association studies (5), but has also been adapted to model transcriptomic investigations (6). To this end, we use λ as a measure to appreciate marked differences in transcriptome gene expression following different concentrations of DEX stimulation. Here, λ is defined as the ratio of the median of the empirically observed distribution of the test statistic to the expected median. Under this paradigm, a mean λ of 1 indicates no difference from the expected null distribution, while λ > 1 indicates shifts from the expected null distribution, as evident for both PTSD- and PTSD+ participants following stimulation with 2.5nM, 5nM and 50nM of DEX.

### Gene co-expression network construction and module detection

### Signed co-expression networks were built using WGCNA in R (7). A total of 21,117 genes were used to construct a global network across all samples. These genes were found to be differentially expressed following 2.5nM, 5nM and 50nM of DEX relative to baseline (0nM). Because gene ontology and pathway enrichment analysis based on large lists of differentially expressed genes can be difficult to perform and interpret, WGCNA was used to facilitate and streamline functional annotation of DEX-responsive genes. To construct a network, the absolute values of Pearson correlation coefficients were calculated for all the possible gene pairs and resulting values were transformed using a β-power of 9 so that the final correlation matrix followed an approximate scale-free topology. The WGCNA cut-tree hybrid algorithm was used to detect sub-networks, or co-expression modules, within the global network, optimizing minimum module size to 50, deep split of five and a tree-cut height of 0.999 to merge neighboring network modules with similar expression profiles. A singular value decomposition of each module’s expression matrix was determined and the resulting module eigengene (ME), equivalent to the first principal component, was used to represent the overall expression profiles for each module. Modules with highly similar ME values were merged into one module (R > 0.75). A one-way ANOVA was used to determine which ME values were dynamically regulated and significantly responsive to DEX across 2.5nM, 5nM and 50nM. MEs were also used to determine module membership (kME) values for each gene in a specified module, defined as the correlation between gene expression values and ME expression. Genes with the highest intramodular kME were labeled as hub genes and predicted to be essential to the function of the module.

### Co-expression and gene set preservation analysis

### To identify gene co-expression modules that are either disrupted or created in response to DEX in PTSD, a permutation-based preservation statistic (Z_summary_) (8) implemented within WGCNA was used. A preservation statistic (Z_summary_) with 200 random permutations was used to measure the (dis)similarity in correlation patterns for the genes within these gene sets, whereby Z_summary_ > 10 indicates strong evidence of preservation, 2 < Z_summary_ < 10 indicates weak-to-moderate evidence of preservation and Z_summary_ < 2 indicates minimal-to-no evidence of preservation. Notably, this analytical framework was first applied in an unsupervised fashion to measure large differences in gene co-expression patterns, as being disrupted or created in PTSD+ relative to PTSD- participants (and vis-versa), following DEX treatment. However, we observed strong preservation statistics between the two groups indicating similar fundamental gene co-regulation within PTSD+ and PTSD- participants (Figure S6), suggesting that major changes in the underlying gene-gene connectivity do not provide a basis for differential transcriptional responses to DEX in PTSD. As a result, we focused our analysis on discrete *a priori* defined collections of genes with shared functional annotations (*i.e.* GO-terms). A weighted correlation network adjacency matrix was defined between the genes for each gene set for PTSD- and PTSD+ participants separately and preservation was examined as previously described.

### Functional enrichment analyses

### All differentially expressed genes with a *P*-value <0.05, after correction for multiple testing, and all network modules with genes showing kME>0.60 were subjected to functional annotation. The ToppFunn module of ToppGene Suite software (https://toppgene.cchmc.org/) (9) was used to assess enrichment of Gene Ontology (GO)-terms relevant to cellular components, molecular factors, biological processes and metabolic pathways using a one-tailed hyper-geometric distribution with a Bonferroni correction. A minimum of a five-gene overlap per gene-set was necessary to be allowed for testing.

### ChIP-Seq overlap analysis

To assess whether DEX-stimulated gene co-expression modules contained a significant enrichment of genes harboring glucocorticoid binding sites, we tested each module for enrichment of human GR binding sites via glucocorticoid response elements using previously generated ChIP sequencing (ChIP-seq) data from A549 cells (**10**). Specifically, these are GR binding sites that are those, which are known to significant gene regulatory activity following glucocorticoid stimulation. In order to assess overlap with these binding sites, genomic coordinates were defined as the start and end positions for each gene (analogous to gene length) within each module. A permutation-based approach with 1,000 random permutations was used to determine statistical significance of the overlap between genomic coordinates for genes with glucocorticoid binding sites using the R package regioneR (**11**). These results should be interpreted with some caution as the effects of glucocorticoid activity have been reported to be tissue specific, and the GR binding sites with gene regulatory potential examined here are those derived from A549 cells and not from peripheral blood tissue.

**Collection of glucocorticoid regulatory genes**

### A collection of 82 genes with known glucocorticoid regulatory actions were downloaded from the Pathway Interaction Database (PID no. M115) and manually curated. The PID (http://pid.nci.nih.gov) is a freely available collection of curated and peer-reviewed pathways composed of human molecular signaling and regulatory events and key cellular processes (12). A total of seven genes were removed on the basis of not being above the level of detection in the current experiment, resulting in a total of 75 glucocorticoid regulatory genes, which were subjected to down-stream analyses. Supplemental Table 5 contains a list of all genes.

### Data availability

### To promote the exchange of this information, we developed an interactive web application with an easily searchable interface to act as a companion site for this manuscript (https://breenms.shinyapps.io/DEXPTSD/). Here users can search for any gene of interest and visualize the dose-dependent effects of DEX on gene expression profiles for trauma-exposed combat veterans with and without PTSD.

### Code availability

### All computational code is available by request to the corresponding author. Alternatively, users can download the code used for this study from the following URL webpage: https://github.com/BreenMS.

### Real-time quantitative PCR

### RT-qPCR was performed to validate gene expression results using five PTSD+ and five PTSD- subjects. Table below is the qPCR primers which were purchased from Thermo Fisher. GAPDH and B2M were used as reference genes. qPCR reagent is KAPA PROBE FAST qPCR Kit from Sigma. Data analysis for qPCR was performed using EasyqpcR R package (13).

| **TaqMan probes used to validate RNA-sequencing expression.** | | |
| --- | --- | --- |
| **Gene Symbol** | Gene Name | Assay ID |
| *LEFTY1* | left-right determination factor 1 | Hs00764128_s1 |
| *IFIT1B* | interferon induced protein with tetratricopeptide repeats 1B | Hs01128267_s1 |
| *CCL25* | C-C motif chemokine ligand 25 | Hs00608373_m1 |
| *GPR88* | G protein-coupled receptor 88 | Hs03027832_s1 |
| *FAM25E* | family with sequence similarity 25 member E | Hs05626253_g1 |
| *GHSR* | growth hormone secretagogue receptor | Hs00269780_s1 |
| *MDK* | midkine (neurite growth-promoting factor 2) | Hs00171064_m1 |
| *OR6C75* | olfactory receptor family 6 subfamily C member 75 | Hs02340385_s1 |
| *OR1N2* | olfactory receptor family 1 subfamily N member 2 | Hs02338872_s1 |

**REFERENCES**

1. Ritchie, Matthew E., et al. "limma powers differential expression analyses for RNA-sequencing and microarray studies." Nucleic acids research 43.7 (2015): e47-e47.
2. Newman, Aaron M., et al. "Robust enumeration of cell subsets from tissue expression profiles." Nature methods 12.5 (2015): 453.
3. Hoffman, Gabriel E., and Eric E. Schadt. "variancePartition: interpreting drivers of variation in complex gene expression studies." BMC bioinformatics 17.1 (2016): 483.
4. Rinne, Thomas, et al. "Hyperresponsiveness of hypothalamic-pituitary-adrenal axis to combined dexamethasone/corticotropin-releasing hormone challenge in female borderline personality disorder subjects with a history of sustained childhood abuse." *Biological psychiatry* 52.11 (2002): 1102-1112.
5. Devlin, Bernie, and Kathryn Roeder. "Genomic control for association studies." Biometrics 55.4 (1999): 997-1004.
6. van Iterson, Maarten, Erik W. van Zwet, and Bastiaan T. Heijmans. "Controlling bias and inflation in epigenome-and transcriptome-wide association studies using the empirical null distribution." Genome biology 18.1 (2017): 19.
7. Zhang, Bin, and Steve Horvath. "A general framework for weighted gene co-expression network analysis." Statistical applications in genetics and molecular biology 4.1 (2005).

1. Langfelder, Peter, et al. "Is my network module preserved and reproducible?." PLoS computational biology 7.1 (2011): e1001057.
2. Chen, Jing, et al. "ToppGene Suite for gene list enrichment analysis and candidate gene prioritization." Nucleic acids research 37.suppl_2 (2009): W305-W311.
3. Vockley, Christopher M., et al. "Direct GR binding sites potentiate clusters of TF binding across the human genome." Cell 166.5 (2016): 1269-1281.
4. Gel, Bernat, et al. "regioneR: an R/Bioconductor package for the association analysis of genomic regions based on permutation tests." Bioinformatics 32.2 (2015): 289-291.
5. Schaefer, Carl F., et al. "PID: the pathway interaction database." Nucleic acids research 37.suppl_1 (2008): D674-D679.
6. Pabinger, Stephan, et al. "A survey of tools for the analysis of quantitative PCR (qPCR) data." Biomolecular Detection and Quantification 1.1 (2014): 23-33.
