# Supplementary material for: Differential transcriptional response following glucocorticoid activation in cultured blood immune cells: a novel approach to PTSD biomarker development
Source: Transl Psychiatry. 2019 Aug 21;9:201. doi: 10.1038/s41398-019-0539-x (PMC6704073; doi:10.1038/s41398-019-0539-x)
Supplement: Supplementary file 2 — Supplemental Figures 1-7 [file 41398_2019_539_MOESM2_ESM.pdf]

Differential transcriptional response following glucocorticoid activation in cultured blood immune cells: a novel approach to PTSD biomarker development

AUTHORS: Michael S. Breen*[1,2,3], Linda Bierer[1,4,5], Nikolaos P. Daskalakis[6], Heather N. Bader[4,5], Iouri Makotkine[1,4,5], Mitali Chattopadhyay[1,4,5], Changxin Xu[1,4,5], Ariela Buxbaum Grice[1], Anna S. Tocheva[7], Janine D. Flory [1,4,5], Joseph D. Buxbaum[1,2,3,8], Michael J. Meaney[9,10], Kristen Brennand[2,8,11,12], Rachel Yehuda*[1,4,5]

AFFILIATIONS: [1]Department of Psychiatry, [2]Department of Genetic and Genomic Sciences, [3]Seaver Autism Center for Research and Treatment, Icahn School of Medicine at Mount Sinai, New York, New York, 10029, USA; [4]Division of Traumatic Stress Studies, Icahn School of Medicine at Mount Sinai, New York, New York, 10029, [5]Mental Health Care Center, James J. Peters Veterans Affairs Medical Center, Bronx, New York, USA; [6]Department of Psychiatry, McLean Hospital, Harvard Medical School, Belmont, MA; [7]Columbia Center for Translational Immunology, Columbia University Medical Center, New York, NY 10032, USA; [8]Friedman Brain Institute, Icahn School of Medicine at Mount Sinai, New York, NY, 10029, USA; [9]Douglas Mental Health University Institute, McGill Group for Suicide Studies, [10]Ludmer Centre for Neuroinformatics and Mental Health, Department of Psychiatry, McGill University, Montreal, Quebec, H3A 0G4, Canada; [11]Icahn Institute of Genomics and Multiscale Biology, [12]Department of Neuroscience, Icahn School of Medicine at Mount Sinai, New York, NY, 10029, USA.

Correspondence to: michael.breen@mssm.edu and Rachel.Yehuda@va.gov

Supplemental Figures 1-7, in brief:

Supplemental Figure 1. Selection of DEX dosage.
Supplemental Figure 2. Quantifying known sources of gene expression variation.
Supplemental Figure 3. Baseline (0nM DEX) differences in gene expression profiles.
Supplemental Figure 4. Unsupervised WGCNA analysis.
Supplemental Figure 5. ChIP-Seq enrichment analysis.
Supplemental Figure 6. Module preservation analysis.
Supplemental Figure 7. Differential gene expression overlap analysis.

Figure S1

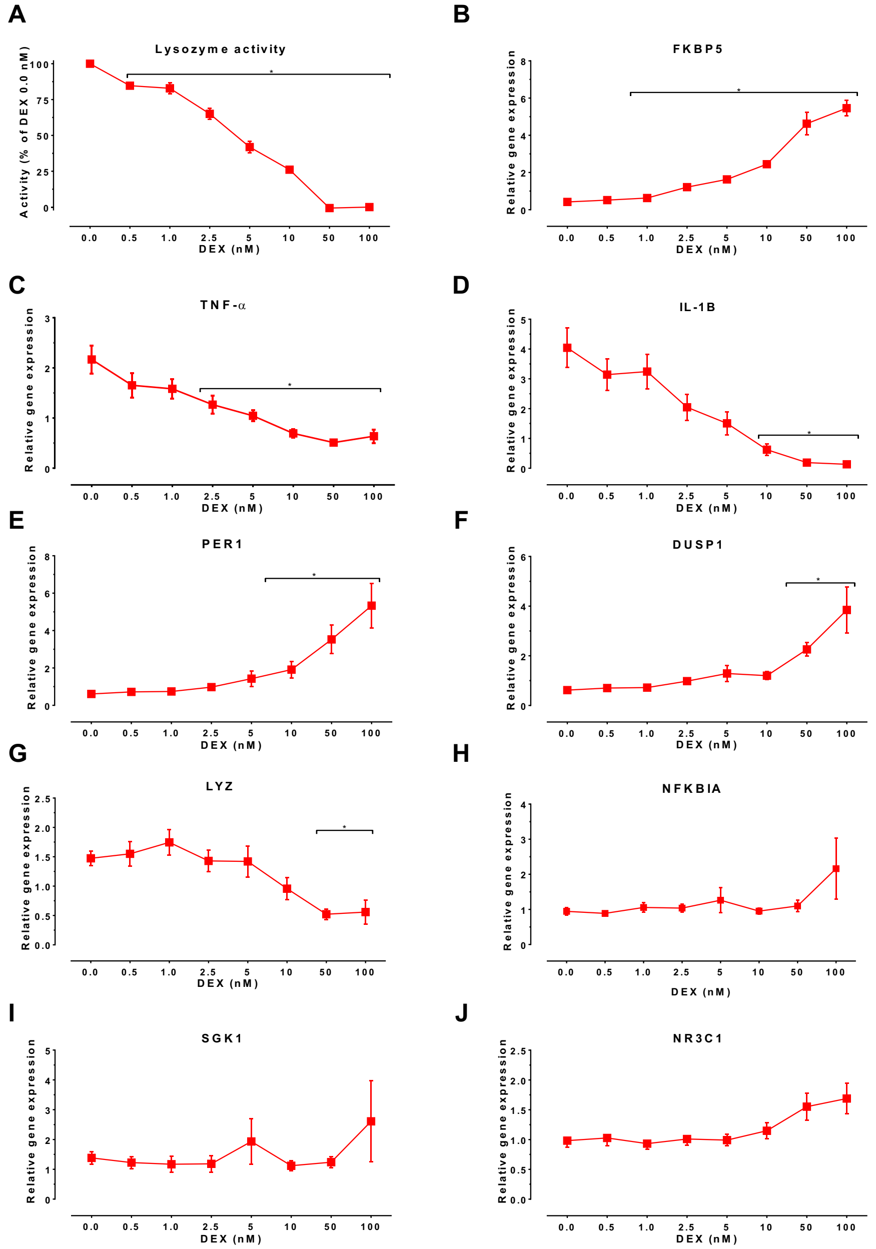

Figure S1. Selection of dexamethasone concentration. (**A**) Lysozyme synthesis is inhibited by DEX, and reduction of lysozyme activity in our system reflects functional responsiveness to glucocorticoids. (B-G) 0 out of 9 genes respond to vehicle, 0 out of 9 genes respond to 0.5nM, 1 out of 9 genes respond to 1nM DEX (FKBP5), 2 out of 9 genes respond to 2.5nM (FKBP5 and TNFα), 2 out of 9 genes respond to 5nM (FKBP5 and TNFα), 4 out of 9 genes respond to 10nM (FKBP5, TNFα, IL-1B, PER1), 6 out of 9 genes respond to 50nM (FKBP5, TNFα, IL-1B, PER1, DUSP1, LYZ) and 6 out of 9 genes respond to 100nM (FKBP5, TNFα, IL-1B, PER1, DUSP1, LYZ). Gene names: Dual specificity protein phosphatase 1 (DUSP1), FK506 binding protein 5 (FKBP5), Interleukin 1 beta (IL-1β), Lysozyme (LYZ), Nuclear factor of kappa light polypeptide gene enhancer in B-cells inhibitor, alpha (NFKBIA), Nuclear receptor subfamily 3, group C, member 1 (NR3C1), Period circadian protein homolog 1 (PER1), Serine/threonine-protein kinase (SGK1), and Tumor necrosis factor alpha (TNFα) (compared to 3 reference genes).

Figure S2

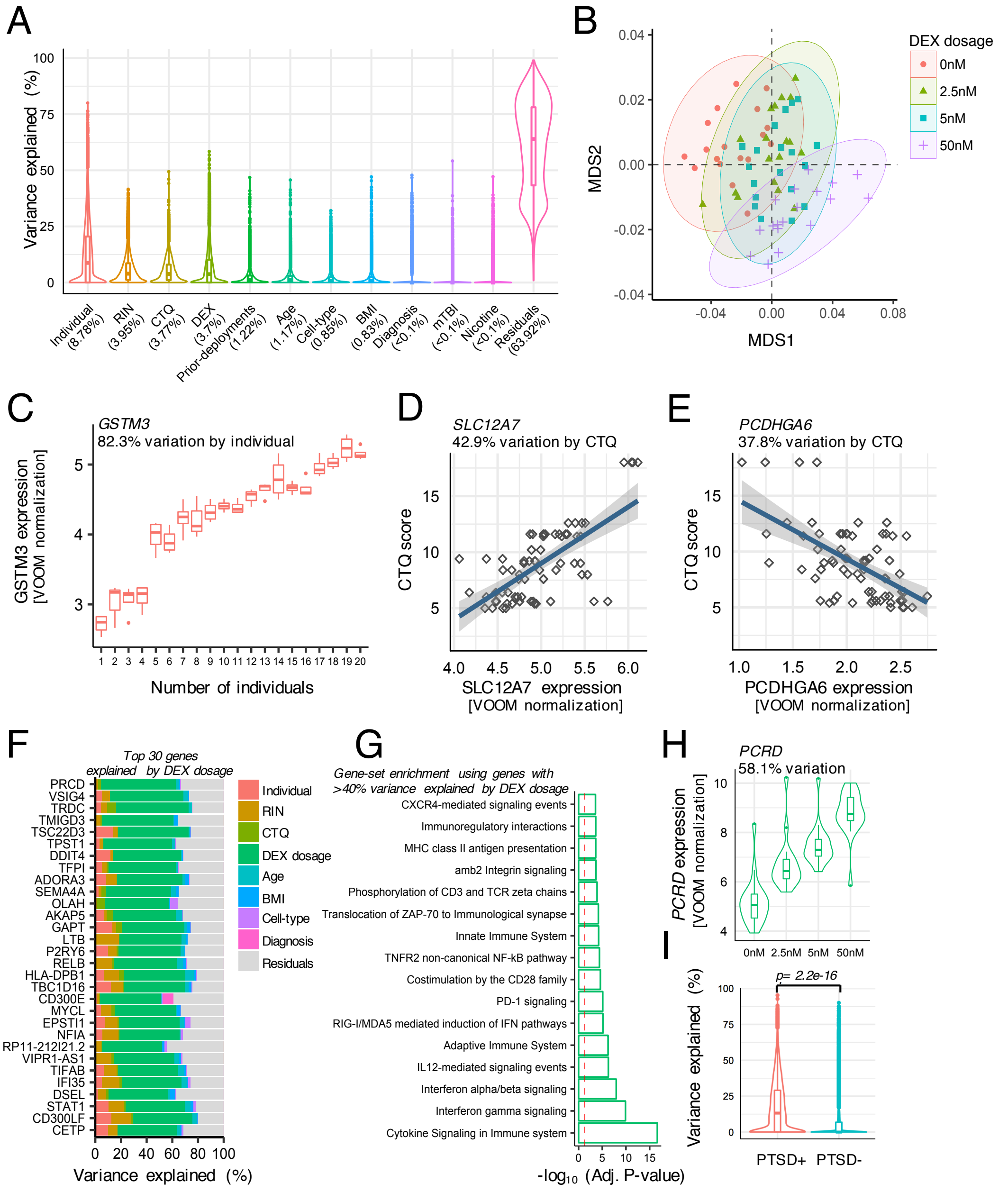

Figure S2. Quantifying known sources of gene expression variation. (**A**) Percentage of gene expression variance explained according to 11 covariates, which represent potential technical and biological sources of variability. We used the linear mixed model framework of the varianceParition R package to quantify variability explained across all available samples. Individual as a repeated measure explains the largest amount of variability in the transcriptome data. (**B**) Principal component analysis further quantified variance in transcriptome-wide data explained by increasing concentrations of DEX. Ellipse's are centered around the mean of each concentration of DEX and wrapped with 95% confidence intervals. (**C-H**) Expression of representative genes stratified by a variable that explains a substantial fraction of the expression variation. (**C**) GSTM3 is a stratified by individual as a repeated measure. (**D**) SLC12A7 and (**E**) PCDHGA6 stratified by childhood trauma questionnaire (CTQ). (**F**) The top 30 genes whose expression variation is explained by different concentrations of DEX. (**G**) Functional enrichment analysis of all genes with >40% variance explained by DEX dosage. (**H**) PCRD stratified by DEX dosage. (**I**) Variance of gene expression explained across dosages for PTSD+ and PTSD- participants separately. Wilcox-rank sum test was used to assess significance.

Figure S3

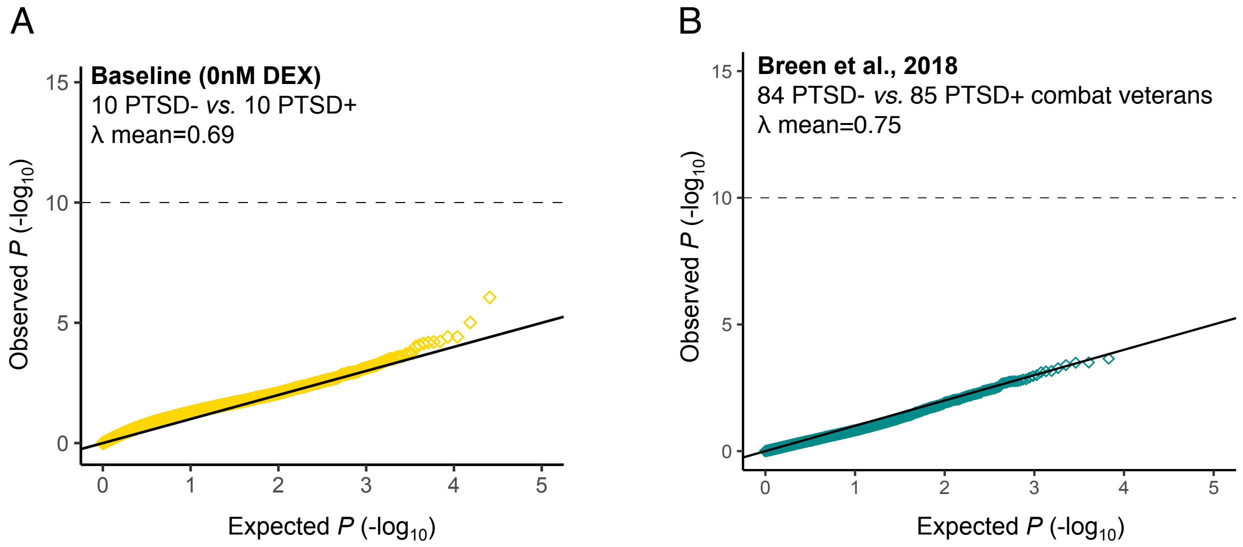

Figure S3. Baseline (0nM DEX) differences in gene expression profiles. (**A**) Differential gene expression compared 10 PTSD- vs. 10 PTSD+ participants at baseline (0nM DEX) and a QQ-plot visualized the distribution of PTSD-associated *p*-values. Lamda (λ) indicates a p-value distribution no different from the expected null distribution, indicative of small effect sizes. (**B**) To confirm this result, we leveraged differentially expressed genes from the largest PTSD blood transcriptome study conducted to date (October 1, 2018) in combat veterans and found a similar distribution of *P*-values, supporting the notion that baseline gene expression profiles often produce small effect sizes and this is likely one obstacle hindering the development of clinically meaningful diagnostic biomarkers.

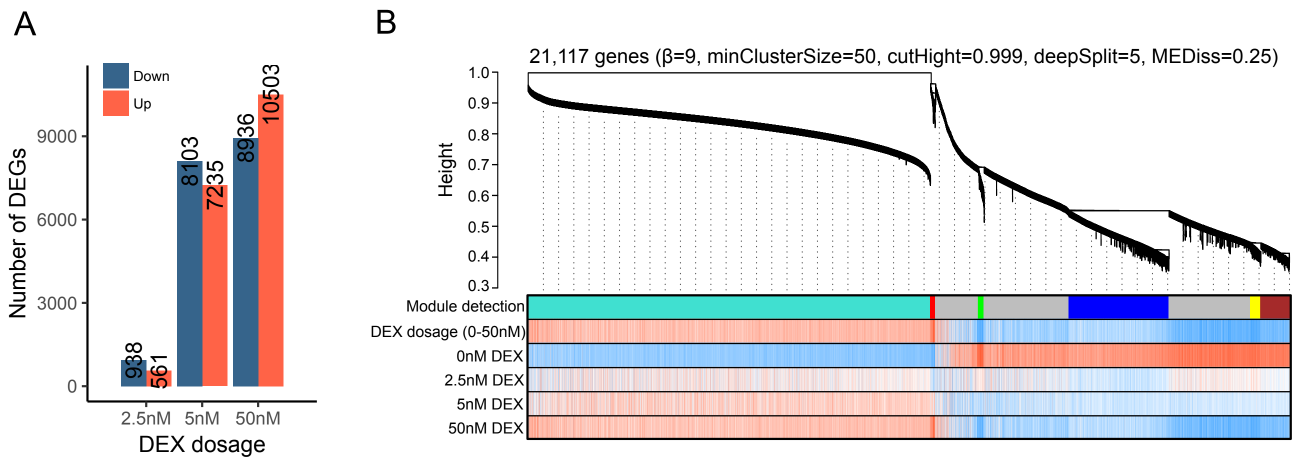

Figure S4 Unsupervised WGCNA analysis. (**A**) Differential gene expression identified a total of 21,117 genes covaring for individual, RIN, childhood trauma, age, prior-deployments, BMI, basal cell type frequencies and differences in PTSD status. This test identified genes that were independent of any PTSD effects. (**B**) These genes were used to construct a weighted gene co-expression network, in order to identify discrete groups of co-regulated genes (modules) that could be better interrogated for their functional responses to DEX. Hierarchical cluster tree (dendrogram) of the combine PTSD+ and PTSD- network is displayed. Each line represents a gene (leaf) and each low-hanging cluster represents a group of co-expressed genes with similar network connections (branch) on the tree. The first band underneath the tree indicates the seven detected, and subsequently analyzed, network modules. The second-sixth bands indicates gene-trait correlations, whereby a red line indicates a strong positive association and a blue line indicates a strong negative association for a particular gene. For a comprehensive functional annotation of each module and calculation of all significant module-trait relationships see **Figure 1**.

Figure S5

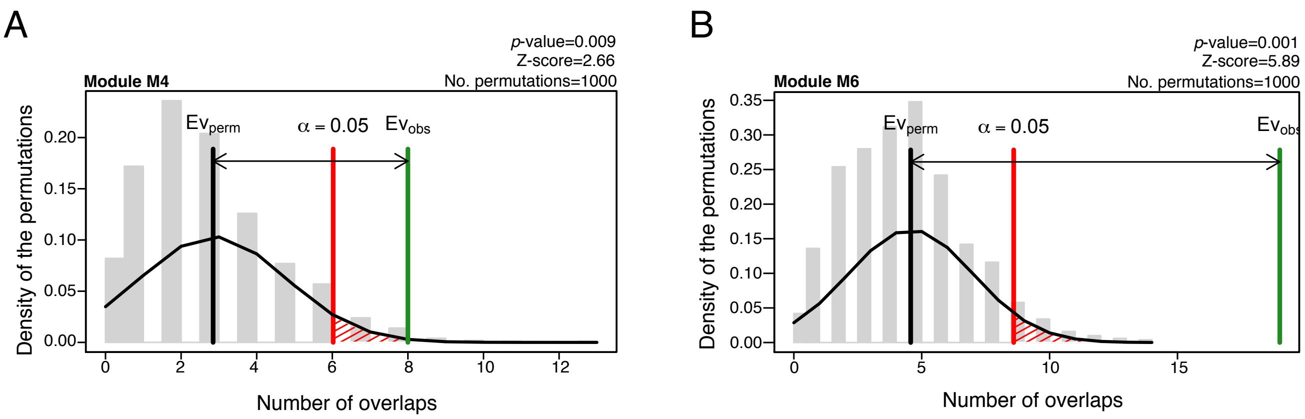

Figure S5. ChIP-Seq enrichment analysis. Genomic coordinates for all genes within each DEX-stimulated gene co-expression module were assessed for enrichment for human glucocorticoid binding sites reported to have known gene regulatory effects in human A549 cells. The regioneR R package was used test overlaps of genomic regions based on permutation sampling. We repeatedly sampled random regions from the genome 1000 times, matching size and chromosomal distribution of the region set under study. By recomputing the overlap with glucocorticoid binding sites in each permutation, statistical significance of the observed overlap was computed. We observed significant enrichment for modules (**A**) M4 and (**B**) M6 with glucocorticoid binding. No significant enrichments were observed for any other module (not shown).

Figure S6

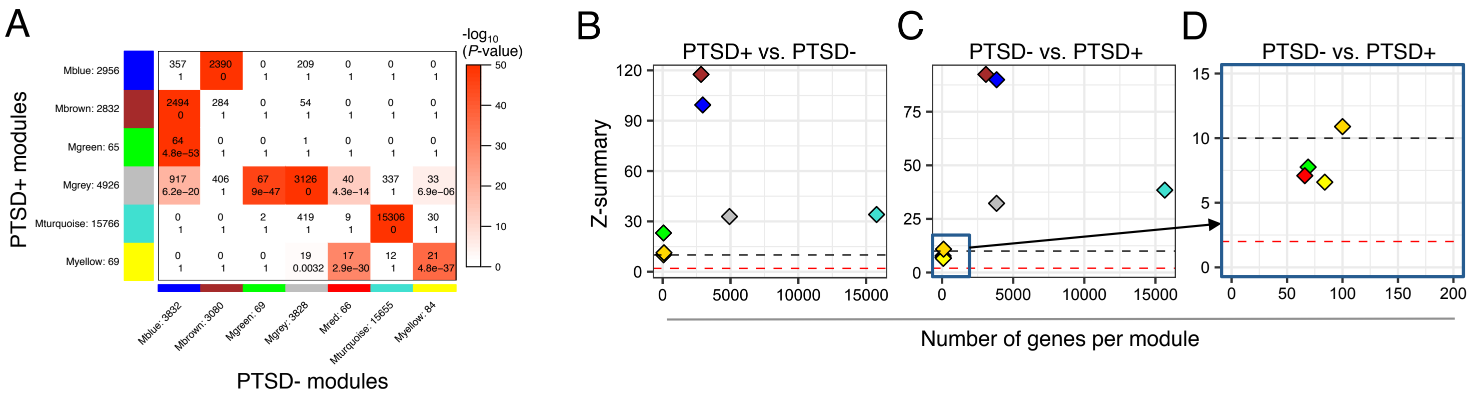

Figure S6. Module preservation analysis. (**A**) Unsupervised WGCNA analysis was applied to PTSD+ participants (y-axis) and PTSD- participants (x-axis) using all genome-wide gene expression data. The extent of module overlap was examined for modules identified within each group. The top value in each cell indicates the total number of overlapping genes and the bottom value indicates the significance of the overlap, computing using a Fisher's exact test. Each cell is color coded by significance (bright red indicates strong significance and white indicates not significant). (**B**) PTSD+ modules were examine for gene co-expression preservation within PTSD- samples. (**C-D**) PTSD- modules were examined for gene co-expression preservation within PTSD+ samples. (**D**) A zoomed scatterplot of three modules (red, green and yellow). Gold modules indicate a random permutated collection of 100 genes. A Zsummary <2 would indicate minimal-to-no module preservation and a differential transcriptional response to DEX that is unique in one group. However, all modules displayed moderate-to-high levels of preservation between PTSD- and PTSD+ participants.

Figure S7

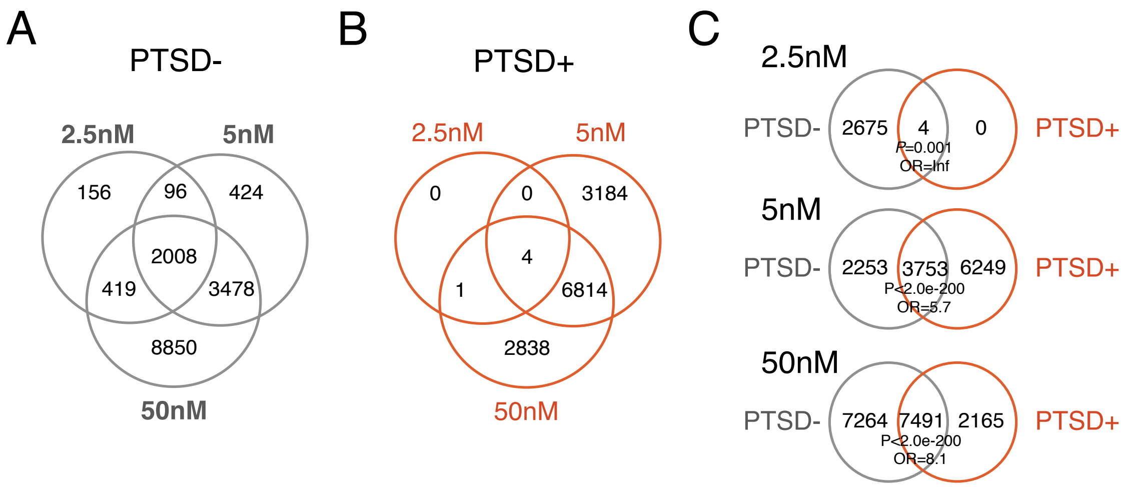

Figure S7. Differential gene expression overlap analysis. DEX-stimulated changes in gene expression were compared across 2.5nM, 5nM and 50nM for (**A**) PTSD- and (**B**) PTSD+ participants. (**C**) We also directly compared PTSD- to PTSD+ for similarities in DEX-stimulated response for each concentration, separately. A Fisher's exact test and an estimated odds-ratio are reported for significant overlaps.
